# Research on the Timing of Replacing Worn Milling Cutters by Using Wear Transition Percentage Constructed Based on Spindle Current Clutter Signals

**DOI:** 10.3390/s25092869

**Published:** 2025-05-01

**Authors:** Zhihao Liu, Min Wang, Zhishan Wang, Tao Zan, Xiangsheng Gao, Peng Gao

**Affiliations:** College of Mechanical & Energy Engineering, Beijing University of Technology, Beijing 100124, China; lzh-zl007@bjut.edu.cn (Z.L.); wang-zhishan@hotmail.com (Z.W.); zantao@bjut.edu.cn (T.Z.); gaoxsh@bjut.edu.cn (X.G.); gaoqindao@bjut.edu.cn (P.G.)

**Keywords:** current clutter, tool wear, convolutional neural network, deep learning, Vold–Kalman filter

## Abstract

The use of worn cutters not only reduces the machining accuracy but also increases the surface roughness. Therefore, it is important for enterprises to establish replacement rules for worn cutters. However, traditional wear regression studies require frequent shutdowns to measure tool wear as training samples. This undoubtedly increases the complexity of operations, making it difficult to apply in practical production. To address this issue, a novel method based on the wear transition percentage has been proposed to determine the optimal timing of replacing worn tools. This method does not require measuring tool wear and is suitable for different machining parameters. Firstly, the Vold–Kalman filter is employed to remove the rotation frequency and its harmonic components from the spindle current, resulting in spindle current clutter signals (SCCS) with low correlation with cutting parameters. Then, using convolutional neural networks (CNN) to learn the SCCS data features of severe wear and normal wear stages, a binary classification CNN model is obtained. Finally, the model is used to identify the full life SCCS data with different cutting parameters. The proportion of samples identified as normal wear to all samples during a certain period of time is used to calculate the wear transition percentage. The effectiveness of this method is verified by comparing it with the measured flank wear.

## 1. Introduction

In milling, the high-speed rotating milling cutter directly contacts the workpiece, generating friction and a large amount of heat while removing material [1]. This process invariably results in tool wear, the extent of which accumulates over time. When wear is too severe, it can increase the cutting force and vibration, leading to a decrease in machining quality and even more serious consequences such as tool breakage [2,3]. If the tool can be replaced in a timely manner within the normal wear limit, it can minimize unnecessary downtime and greatly improve production efficiency. Therefore, tool condition monitoring is one of the research hotspots in intelligent manufacturing, and it is also an urgent technology needed by production enterprises.

The direct and indirect methods are two commonly used tool wear monitoring methods. The former requires stopping the processing first and then evaluating the degree of wear using optical equipment and machine vision technology [4,5]. This type of method has high accuracy, but it increases working hours and cannot achieve real-time health monitoring. Therefore, the indirect method, which can be monitored online, has garnered significant attention from scholars in the field. The indirect method is a data-driven approach. Sensor signals related to tool wear are acquired and used for wear assessment during the machining process. The physical signals commonly used for tool health monitoring include cutting force [6,7], acoustic emission (AE) [8,9], vibration acceleration [10,11,12], temperature, and current [13,14]. Liu et al. [15] measured the cutting temperature and force, and used a flank wear rate model to calculate and correct the flank wear width. Benkedjouh et al. [16] collected AE, vibration acceleration, and force signals during the machining process and proposed a methodology for tool condition evaluation based on support vector regression and nonlinear feature reduction. Li and Tso [17] used regression analysis to study the correlation between machining parameters and current signals. Then, the wear status of the tools was identified through fuzzy classification methods. In summary, vibration, AE, and cutting force signals can efficiently and accurately reflect tool wear, but their limitations make it difficult to apply them on a large scale in actual machining. Firstly, collecting these signals requires installing sensors in the processing area, which not only interferes with normal processing but also limits the shape and size of the workpiece. Secondly, the price of platform dynamometers, acceleration sensors, and their signal amplification equipment is expensive. Most factories would rather continue to use traditional tool-changing strategies than pay for expensive tool wear monitoring systems. However, Hall current sensors do not have the above-mentioned problems, they will not affect processing, and the price is not expensive. It has the prospect of large-scale promotion and application in factories.

The waveform of the spindle current signal is affected by cutting parameters [18,19]. For example, the frequency and amplitude of the current signal are affected by spindle speed. Similarly, changes in feed depth can cause changes in cutting load, ultimately leading to an increase or decrease in current amplitude. Therefore, directly using the unprocessed spindle current signal to detect the status of the cutters is not suitable for different processing conditions. The universality of the tool health level monitoring system will be limited. Song et al. [20] pointed out in their study that the raw spindle current signal is composed of a clutter signal and a fundamental signal. The latter is composed of the spindle rotation frequency and its harmonic components, which are affected by the quasi-static cutting force. Therefore, it is highly correlated with cutting process parameters. The clutter signal in the current is caused by dynamic force, with low correlation with cutting parameters and high correlation with tool wear. Therefore, in order to achieve a wear monitoring method that can adapt to different processing parameters, the spindle current clutter signals (SCCS) should be derived by subtracting the fundamental signal from the raw spindle current. To obtain SCCS, Song et al. [20] used a Fourier series to fit the fundamental signal and subtract it from the raw signal. There is a shortcoming in using this method to extract SCCS: because the spindle speed may fluctuate slightly during the machining process, there is a certain bandwidth in the frequency domain for the rotational frequency and its harmonics. The fourth-order Fourier series cannot perfectly eliminate the fundamental signal from the original signal, so a small amount of cutting parameter-related components is retained in SCCS. The Vold–Kalman filter will be employed to address this problem in this study.

The convolutional neural network (CNN) model, which is representative of deep learning algorithms, has achieved prominence in artificial intelligence applications such as computer vision [21]. In comparison with conventional machine learning algorithms, its primary advantage lies in its capacity to extract features from high-dimensional raw data by means of training [22]. This process is called feature learning [23]. Due to CNN’s substantial advantages in regression modeling and pattern recognition, scholars have started to employ it for tool wear monitoring [24,25]. Aghazadeh et al. [26] used spectral subtraction algorithms and wavelet time-frequency transformation to extract features of force, vibration, and current signals. They then employed a CNN to predict wear width.

In addition, many improved models or integrated models for tool wear monitoring have been proposed. Zhao et al. [27] proposed a model that integrates CNN and long short-term memory networks. This model is used to process raw sensor signals and complete wear regression. Wang et al. [9] integrated the preprocessor into the ResNet classifier to improve the recognition accuracy of tool wear classification. The preprocessor is constructed by a denoise transformer Auto-Encoder. Wang et al. [1] proposed a novel deep learning architecture based on CNN, which introduces Siamese structures and auxiliary inputs into the model. This method significantly improves the feature extraction and generalization ability of the model, but the efficiency decreases by 44%. Since the purpose of this article is to achieve a low-cost method for determining tool replacement timing, complex models with high computational requirements were not used. In addition, the indicator provided in this study to determine the tool replacement time is the wear transition percentage, rather than being directly based on the recognition results of the classifier. Therefore, it is not required for the classifier to have an absolute high accuracy. For comprehensive considerations of cost efficiency and real-time performance, in this study, a CNN is trained to learn the nonlinear relationship between milling cutter wear status and current clutter.

**Figure 1:**
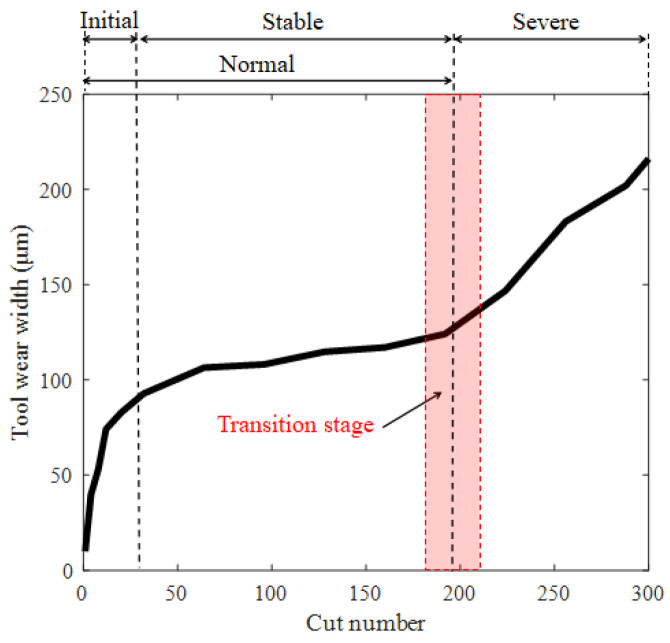
Typical tool wear curve.

A tool wear curve usually includes three stages: initial wear, stable wear, and severe wear [28], as shown in Figure 1. The focus of this study is on determining the timing of replacing worn tools. Since tools in the initial and stable wear stages can be used normally, they are classified as normal wear stages in this study. Usually, the initial wear rate is relatively fast, and it soon enters a stable wear state. During the stable wear phase, the wear width increases slowly. Upon attaining a specific degree of wear, the material of the tool reaches its fatigue limit, thereby entering a phase of severe wear. At this point, the machining accuracy is significantly reduced, and the surface roughness also increases. Therefore, the optimal timing for tool replacement is during the transition stage from stable wear to severe wear. In existing research, both tool wear regression and tool wear classification require measuring tool wear values. In classification research, it is necessary to accurately classify training samples based on wear width. In regression research, wear width is used as the training target. In order to collect a sufficient number of training samples, it is necessary to frequently stop machining to measure tool wear during the model training phase. Therefore, the research results are difficult to apply widely in practical production.

This paper proposes a novel methodology for determining the optimal timing for replacing worn milling cutters. This method is based on SCCS and wear transition percentage, does not require measurement of tool wear, and is suitable for different machining parameters. The Vold–Kalman filter is employed to eliminate the rotation frequency and its harmonic components from the spindle current signal and obtain SCCS with low correlation with cutting parameters. Then, CNN is trained into a binary classification model using the SCCS images from normal wear and severe wear stages. The full life SCCS data are divided into multiple groups in chronological order, and the development trend from normal to severe wear is determined based on the proportion of samples identified as normal wear in each group. The optimal time to replace the worn milling cutter is determined accordingly.

The rest of the paper is organized as follows: Section 2 introduces the proposed methods. Section 3 describes the experimental setup in detail. Section 4 presents the completed experiment. The robustness of the method and the limitations of the current study are discussed in Section 5. Lastly, Section 6 concludes the paper.

## 2. Proposed Method

**Figure 2:**
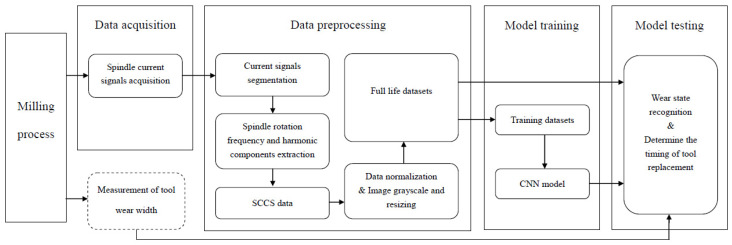
The framework of the proposed method.

Figure 2 shows the framework of the proposed method for determining the timing of replacing worn milling cutters based on SCCS and CNN. It mainly includes the collection of spindle current signals, the extraction of SCCS data, the training of CNN models, the recognition of wear status, and the determination of tool replacement timing.

The implementation steps of this method are as follows:

Step 1: Data acquisition: The Hall current sensor is installed on a cable of the spindle motor of a CNC machine tool to collect real-time raw current signals during the milling process. After completing the milling of each surface, the flank wear of the tool is measured using an industrial camera and a telecentric lens. The number of completed surfaces is recorded as the cut number. Flank wear (VB in μm) refers to the distance from the end of wear on the flank face to the cutting edge [26]. It should be stated here that measuring flank wear is only used to validate the feasibility of the method, but it is not necessary in practical applications.
Step 2: Data preprocessing: Firstly, the raw current is segmented into a multitude of samples. Each segmented sample only contains data from one rotation of the spindle. Then, a Vold–Kalman filter is used to extract the spindle rotation frequency and its harmonic components from the segmented samples. These components, highly correlated with cutting parameters, are removed from the spindle current signal, and SCCS data with low correlation with cutting parameters are obtained. Finally, SCCS are normalized and plotted on the image. The image is grayscale and resized.
Step 3: Model training: The training dataset is employed for the training of the CNN, with the error back propagation algorithm utilized to make adjustments to the network parameters that were initially assigned randomly. The training dataset only contains two types of data, normal wear and severe wear, and therefore, this model is a binary classification model. The normal wear dataset includes the first 20 surfaces completed in the full life dataset. The severe wear dataset includes the last 20 surfaces completed in the full life dataset. The optimized CNN has the ability to extract features indicating tool status from SCCS images.
Step 4: Model testing: The full life dataset under different cutting conditions, different from the training dataset, is input into the optimized CNN. The entire process of milling cutter wear is classified, and the complete process of the tool from the sharp state to the passive state is identified. Meanwhile, the wear transition percentage of the cutting tool is calculated, and the timing for replacing the worn milling cutter is determined based on the slowly decreasing wear transition percentage.

### 2.1. Data Preprocessing

This section will provide a detailed introduction to the preprocessing method of spindle current signals, which includes three steps: current signal segmentation, SCCS extraction, and SCCS image generation.

**Figure 3:**
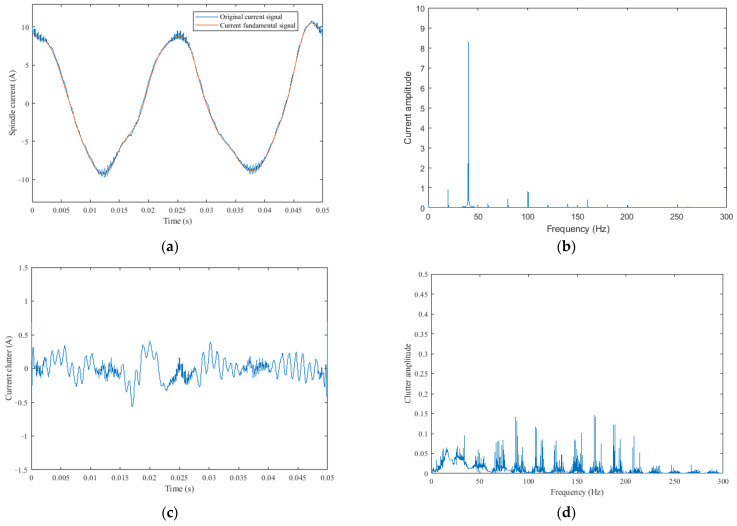
(**a**) The original current signal and fundamental signal, (**b**) frequency spectrum of the current signal, (**c**) current clutter signal, and (**d**) frequency spectrum of the clutter signal.

Step 1: Current signal segmentation: Prior to SCCS extraction, the collected spindle current signal undergoes division into multiple samples. The principle of segmentation is to make each sample only contain data with one rotation of the spindle. This not only reduces the size of each sample but also includes information about all the teeth on the milling cutter. The blue curve in Figure 3a is the original current signal of the spindle rotating one revolution. The spindle speed in Figure 3 is 1200 r/min, the number of pole pairs of the spindle motor is two, and the milling cutter has three teeth.
Step 2: SCCS extraction: Figure 3b shows the spectrum of the raw spindle current. It can be seen that the amplitude of the spindle rotation frequency (20 Hz) and its harmonic components is relatively high and concentrated below 200 Hz. These components form the fundamental wave signal and are related to cutting parameters. Therefore, in order to achieve a wear monitoring method that can adapt to different processing parameters, the SCCS should be calculated by removing the fundamental signal from the raw spindle current. The first method that comes to mind for completing this task is the Fourier technique. However, in Step 1, the current signal has already been segmented, and the Fourier technique has a serious “picket fence effect” when processing short-term signals, which reduces frequency resolution. Increasing the length of the current signal results in a reduction in the number of samples used to calculate the wear transition percentage, which reduces the response rate for wear state identification. Vold and Leuridan [29] proposed a high-resolution Vold–Kalman filter, which is based on the traditional Kalman filter, which can extract and reconstruct time-domain signals of a certain frequency component from raw data and is commonly used for order tracking [30]. Additionally, this method does not have the above-mentioned problems. Therefore, a Vold–Kalman filter is used to obtain the spindle rotation frequency and its first 15 harmonic components from the original current signal. These components are summed to form the fundamental signal, as shown by the orange curve in Figure 3a. More details about the Vold–Kalman method can be found in Vold’s publication.

The SCCS is calculated by subtracting the fundamental signal from raw data, as shown in Figure 3c. The parameters of the Vold–Kalman filter mainly include the order and the weighting factor r of the structural equation. The weighting factor exerts an influence on the bandwidth of the filter [31]. After testing and comparison, when the weighting factor r is 200,000, it can remove the components within a bandwidth of 2.5 Hz around the spindle rotation frequency and its harmonic components, as shown in Figure 3d. Due to the frequency fluctuation of the spindle within this range, the weighting factor r is set to 200,000. This can avoid the impact of spindle speed fluctuations during processing. When using a smaller weighting factor r, it will result in a larger filter bandwidth and remove too many useful frequency domain components. In addition, it was found through testing that the order of the Vold–Kalman filter has little effect on the results, so the order is set to 1.

**Figure 4:**
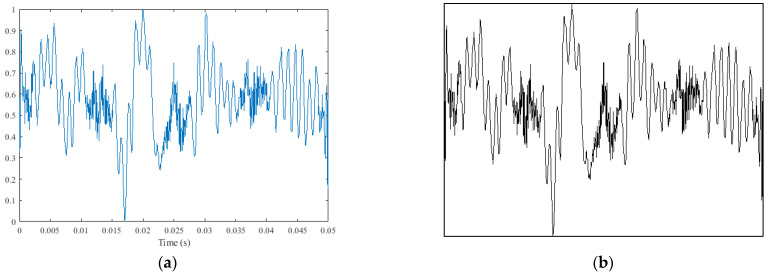
(**a**) Normalized current clutter signal and (**b**) grayscale image of SCCS (The data in the figure comes from Figure 3c).

Step 3: SCCS image generation: As shown in Figure 4a, SCCS was normalized to the range of [0, 1] and plotted on the image. Since the color of the image was not helpful for this study, after normalization, as illustrated in Figure 4b, the image was transformed from a three-channel color image to a grayscale image. This can reduce the amount of data and save the resource cost of neural networks. Finally, the size of the image was adjusted to 160 × 160.

### 2.2. Convolutional Neural Network Model

**Figure 5:**
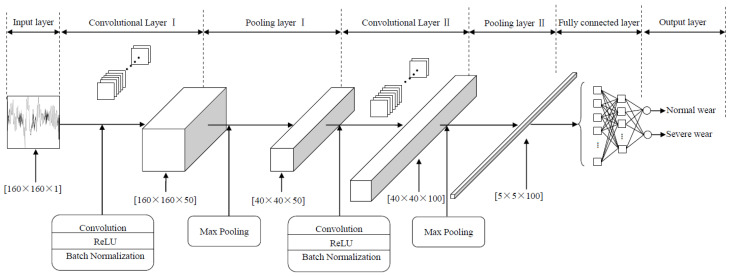
Convolutional neural network structure.

As is well known, CNN has its unique advantages in processing image tasks. Therefore, in this study, a CNN model similar to LeNet was established to learn information about tool wear status from SCCS images. As shown in Figure 5, the CNN model has a total of eight layers. The detailed parameters of the model are shown in Table 1. In addition to the input layer and output layer, it also includes two alternating convolutional and pooling layers, as well as two fully connected layers.

The size of the input layer is 160 × 160 × 1, which corresponds to single-channel SCCS images.

The convolutional layer is a weighted summation process, where the weights are the convolutional kernels. Afterwards, through activation function and batch normalization, the output feature maps are obtained. The general expression for convolutional layers is as follows:

(1) $$x_{j}^{l}=f\left(\sum_{i=1}^{M_{l-1}}x_{i}^{l-1}*k_{ij}^{l}+b_{j}^{l}\right),\;\;\;\;\;\;\;\;j=1,\;2,\;\ldots ,\;M_{l}$$

where f is a nonlinear activation function, ∗ represents the convolution operation, l is used to denote layer index, M is the number of feature maps, $k^{l}$ represents convolutional kernel, $x_{i}^{l-1}$ represents previous layer’s output feature map, and $b^{l}$ represents output feature map’s bias. The activation function in this model is a rectified linear units (ReLU) function. Its expression is as follows:

(2) $$f\left(x\right)=max\left(0,\;x\right)$$

The model is composed of two convolutional layers. The convolution stride size is 1 × 1, and the kernel size is 5 × 5. The number of convolution kernels is 50 and 100, respectively.

The pooling layer is used after the convolutional layer to generate a down-sampled version of the output feature map. This can be understood as the pooling layer being the selection and compression of features extracted by the convolutional layer. The expression for pooling layers is as follows:

(3) $$x_{j}^{l}=down\left(x_{j}^{l-1}\right),\;\;\;\;\;\;\;\;\;\;\;\;\;\;\;j=1,\;2,\;\ldots ,\;M_{l}$$

where down is a pooling function. The pooling function in this model is maximum pooling.

The model is composed of two pooling layers. The pooling kernel of the first layer is 4 × 4, and the stride is 4 × 4. The pooling kernel of the second layer is 8 × 8, and the stride is 8 × 8.

The convolutional layer and pooling layer complete the feature extraction of SCCS images and, finally, complete the classification through fully connected layers and a Softmax function.

After model training, the weights and biases of the CNN are modified to obtain the optimal results. The optimization algorithm used during model training is Adam, with an initial learning rate of 0.01, a maximum epoch of 300, and a batch size of 128.

### 2.3. Calculation of Wear Transition Percentage

As wear is a slowly changing process, the full life dataset is divided into multiple groups in chronological order, with N samples in each group. By calculating the proportion of normal wear identified by CNN in each group, the development trend from normal to severe wear can be determined. The wear transition percentage is calculated as follows:

(4) Percentage=NNorN×100%

where $N_{Nor}$ represents the number of samples recognized by CNN as normal wear in the group, and N denotes the total number of samples in the group.

## 3. Experimental Setup and Dataset

### 3.1. Experimental Setup

**Figure 6:**
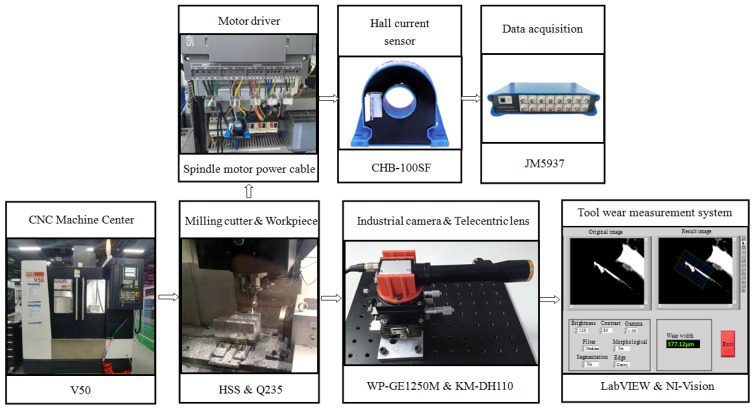
Milling processing equipment and acquisition system.

To verify the effectiveness of the method proposed in this paper for determining the timing of replacing worn milling cutters, multiple milling wear experiments were designed and conducted, with each experiment involving a distinct set of cutting parameters. The experiment was conducted on a V50 vertical machine tool, with the milling cutters and workpieces made of high-speed steel (HSS) and Q235 structural steel, respectively, as illustrated in Figure 6. The diameter of the milling cutters is 16 mm, the number of teeth (edge) is three, and the type is end milling cutter. A Hall current sensor (CHB-100SF, SENSOR Electronics, Beijing, China) is installed on the power cable between the spindle motor (main motion electric motor) and its driver to collect the single-phase current of the main motion electric motor. And the sampling frequency of the data acquisition system is 10 KHz. At the same time, an industrial camera (WP-GE1250M, WORK POWER, Shenzhen, China) and telecentric lens (KM-DH110) are used to measure the wear width of the cutter’s flank face after each surface has been milled. The resolution of the industrial camera is 4072 × 3046, and the pixel size is 1.85 μm × 1.85 μm. When the camera is combined with a telecentric lens, the field of view is 7.5 mm × 5.6 mm, and the real-world size of each pixel is 1.85 μm. This device can capture tool wear bands that appear silver-white in the dark field of view, with a large depth of field, high resolution, and low distortion in the image. The measurement software is developed based on LabVIEW 2014 and the NI-Vision 2014 toolkit, and can complete the acquisition, storage, processing, and wear width measurement of tool images.

The objective of this study was to verify the applicability of the methodology under various working conditions. To that end, three milling cutter wear life experiments with different machining parameters were completed (C1, C2, and C3). They were set with different cut depths in the axial direction (*a_p_*), the cut depths in the radial direction (*a_e_*), feed rates, and spindle speeds. In addition, the number of surfaces they had machined was also different. C1, C2, and C3 had completed milling 116, 156, and 170 surfaces, respectively. The cutting parameters of the milling experiment are listed in Table 2.

### 3.2. Datasets

The datasets contain 88,400 SCCS images of three milling cutters (C1, C2, and C3). Due to the fact that each surface completed by milling can obtain 200 SCCS images, the full life datasets for C1, C2, and C3 contain 23,200, 31,200, and 34,000 SCCS images, respectively. The training datasets for each milling cutter are extracted from its full life datasets. The training dataset includes two types of samples: normal wear (Nor) and severe wear (Sev). The normal wear dataset includes data from the first 20 surfaces of all surfaces completed by a milling cutter. The severe wear dataset includes data from the last 20 surfaces of all surfaces completed by a milling cutter. Therefore, the training dataset for each milling cutter contains 8000 SCCS images.

To test the applicability of the method to various cutting parameters, three CNN models were trained in this paper, namely T1, T2, and T3. Table 3 describes in detail the test dataset and the training dataset used for the training of each model. In the training of the model, the training and testing samples come from different datasets. For example, the T1 model was trained using the training datasets of C2 and C3, and its testing used the training dataset of C1. When determining the timing for replacing the cutter, the trained T1 was used to identify the full life dataset of C1. The proportion recognized by the model as normal wear was calculated to determine whether tool replacement is necessary.

The purpose of designing the experiment in this way is to conduct cross-validation using data from three different cutting parameters. The model can predict the tool state of other unknown cutting parameters by learning data from any two different cutting parameters. In practical applications, even if parameters such as cut depth, spindle speed, or feed rate change after creating the model, there is no need to retrain the model, and the original model can be used to predict tool replacement time.

## 4. Results

### 4.1. Model Training

**Figure 7:**
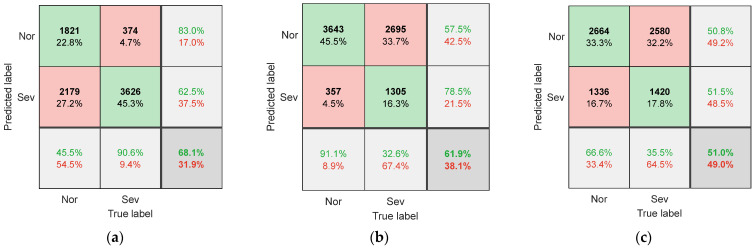
The confusion matrix for (**a**) T1, (**b**) T2, and (**c**) T3 when using the original current signal as input.

**Figure 8:**
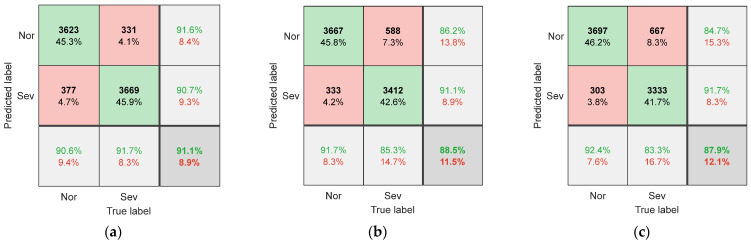
The confusion matrix for (**a**) T1, (**b**) T2, and (**c**) T3 when using the fourth-order Fourier series method to extract SCCS as input.

**Figure 9:**
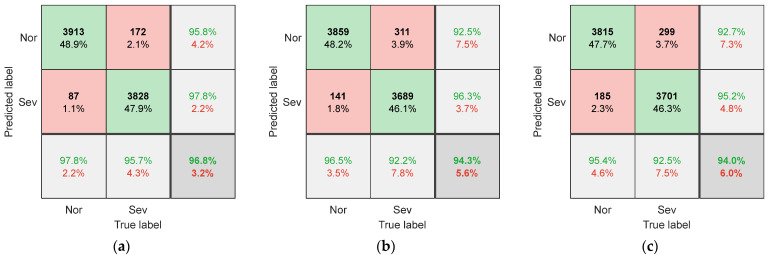
The confusion matrix for (**a**) T1, (**b**) T2, and (**c**) T3 when using the Vold–Kalman filter to extract SCCS as input.

To evaluate the performance of SCCS and its extraction methods, the proposed method was compared with other methods. One comparative experiment used images of the original spindle current signal as input samples, without extracting SCCS from them. Another comparative experiment is to extract SCCS using the fourth-order Fourier series method proposed by Song et al. [20], instead of using a Vold–Kalman filter. Each comparative experiment used the dataset partitioning method introduced in Section 3.2 to obtain the corresponding training and testing sets. Figure 7 shows the confusion matrix of the CNN model on the test set when the raw data image of the current is used as input. The confusion matrix when using the Fourier series method to extract SCCS as input is shown in Figure 8. Figure 9 shows the classification confusion matrix obtained using the method described in this paper.

When using the original spindle current as the input sample, as shown in Figure 7, the model’s recognition accuracy is generally low (below 70%). This is due to the difference in cutting parameters between the testing dataset and the training dataset, resulting in significant differences in the original signal of the current. This phenomenon is most evident in Figure 7c, as the C3 changes the spindle speed, resulting in changes in both the amplitude and frequency of the spindle current. The difference in the original signal is the largest, and therefore, the recognition accuracy is the lowest (51.0%). This indicates that the raw signal of the current contains components highly related to cutting parameters, which are directly used as input samples and are not suitable for situations with variable machining parameters.

As shown in Figure 8 and Figure 9, when using SCCS as input, the model’s recognition accuracy is significantly enhanced. This indicates that the correlation between clutter signals and cutting parameters is low, and there is a high correlation with wear. By training a CNN, it is possible to learn the mapping relationship between SCCS and tool wear status. In addition, compared with the Fourier series method, using a Vold–Kalman filter to extract SCCS results in higher recognition accuracy. The recognition accuracy under three different working conditions reaches 96.8%, 94.3%, and 94.0%. The current signal cannot be perfectly fit by the Fourier series due to fluctuations in the spindle speed during milling. However, the Vold–Kalman filter has a certain bandwidth, which can more effectively remove the spindle rotation frequency and harmonic components from the current signal. This lowers the correlation between the obtained SCCS and cutting parameters, thereby enhancing its suitability for variable working conditions.

**Figure 10:**
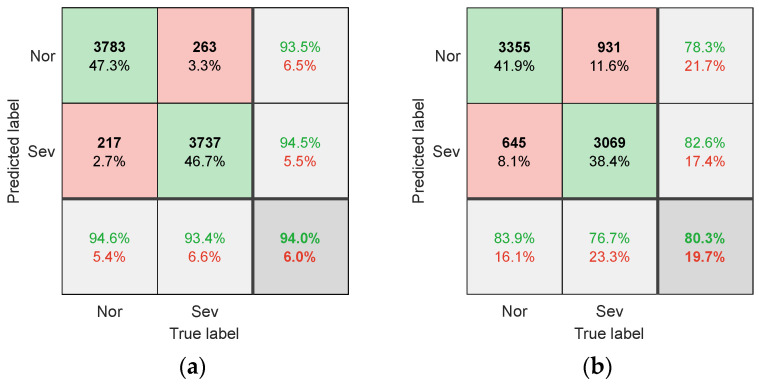
The confusion matrix for (**a**) the 1D-CNN and (**b**) the MLP.

In addition, a comparison experiment with different input forms was conducted. This experiment used the dataset of the T1 model. Three types of neural network models were used, namely multilayer perceptron (MLP), CNN, and one-dimensional CNN (1D-CNN). The 1D-CNN and MLP inputs were scalar data of SCCS, i.e., there were no clutter signals transformed into images. The scalar data size was 500 × 1. The input of the CNN was still SCCS images. The structural parameters of the 1D-CNN were the same as those of the CNN, except that they were changed to one-dimensional. MLP was set as a four-layer neural network, with an input layer containing 500 neurons, two hidden layers containing 60 and 10 neurons, and an output layer containing 2 neurons. The activation functions of all layers were Sigmoid functions. The purpose of this setting was to make it the same as the fully connected layer of the CNN. The confusion matrix when using SCCS images as inputs for the CNN is shown in Figure 9a. The confusion matrix when using SCCS scalar data as input for the 1D-CNN and MLP is shown in Figure 10. The experimental results show that compared with the other two direct signal recognition schemes, using images as input has a higher recognition rate. This is because conventional machine learning algorithms like MLP are more suitable for feature engineering applications, where their input is preferably some feature values rather than raw scalar data. Afterwards, the optimized CNN model was used to identify the full life dataset of cutting tools and determine the timing of replacing worn milling cutters.

### 4.2. Determining the Timing for Tool Replacement

Analyses were conducted in order to accurately identify the transition time from normal wear to severe wear of the milling cutter, that is, to determine the point at which the width of milling cutter flank wear begins to rapidly increase. A CNN prediction model trained on datasets of normal and severe wear stages was used to test the full life dataset of milling cutters. As wear is a slowly changing process, the full life dataset was divided into multiple groups in chronological order, with 200 samples in each group. By calculating the proportion of normal wear identified by CNN in each group, the development trend from normal to severe wear can be determined. The wear transition percentage was calculated using Equation (4).

**Figure 11:**
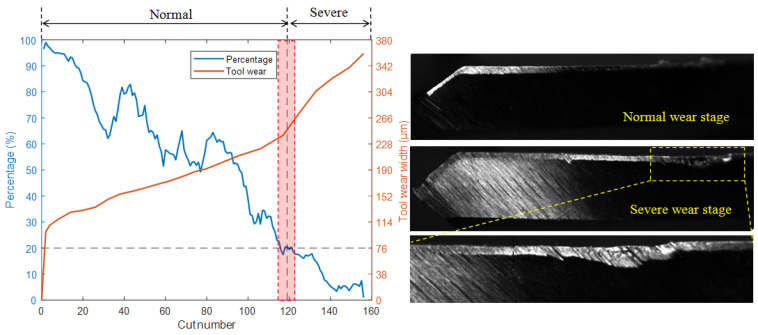
Test results of the T2 model.

Figure 11 shows the test results for the T2 model. The red background area in the figure represents the transition stage, the yellow dashed line represents zoom, and the black dashed line is the auxiliary line. For a clearer comparison, the wear transition percentage (blue) and the tool wear width measured with a camera (orange) are plotted on the same graph. As the cut number increases, the width of tool wear also increases continuously, while the percentage shows a fluctuating downward trend. It was found that when the wear width of the cutter changed from a stable increase to a rapid increase, the percentage was around 21%. From the photo of the flank face in Figure 11, it can be observed that during the normal wear stage, the surface of the wear band is smooth, the width of the wear band is uniform, and the edge of the milling cutter is intact. However, after entering the severe wear stage, the friction and contact between the workpiece and the cutter, as well as the chips, cause an increase in temperature, and the hard points of the material leave small scratches on the surface of the tool. Also, the scratches are parallel to the relative movement direction of the milling cutter, indicating a severe abrasive wear phenomenon at this time. In addition, severe boundary wear was observed on the flank face, on the side near the workpiece surface. At the same time, there is also a small amount of damage on the edge of the cutter. Due to severe wear on the cutting tool at this stage, the roughness of the machined parts increases, and the surface quality and accuracy decrease. In more serious cases, it may cause the milling cutter to collapse and fracture. Therefore, in order to ensure machining quality, tool replacement should be carried out in a timely manner before entering this stage.

**Figure 12:**
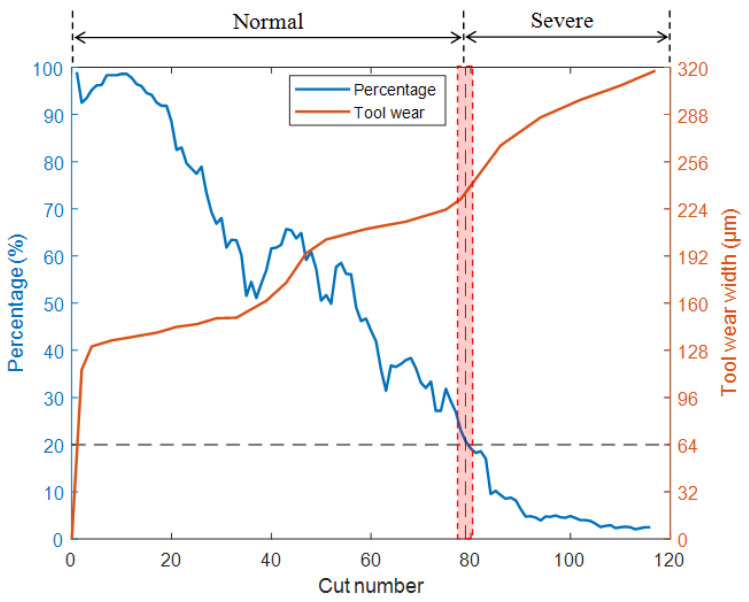
Test results of the T1 model.

**Figure 13:**
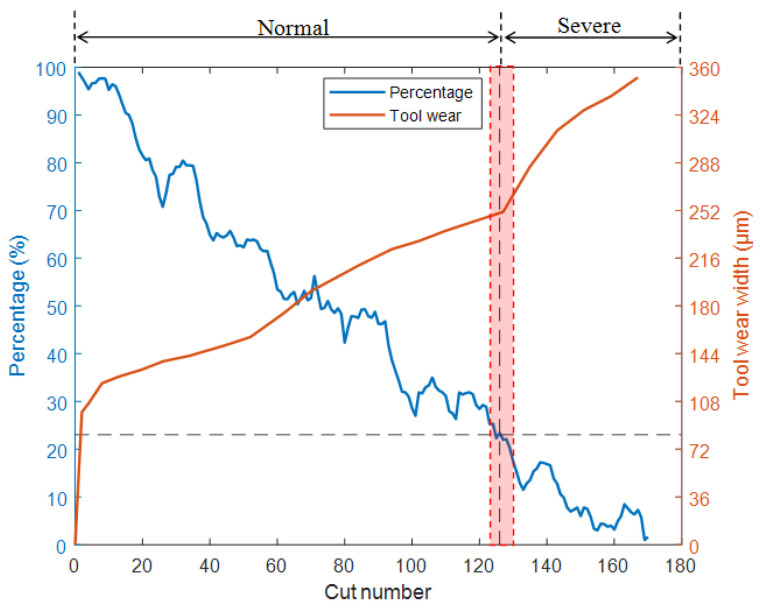
Test results of the T3 model.

As shown in Figure 12 and Figure 13, the test results of T1 and T3 models also indicate that when the cutter wear level is about to enter the stage of severe wear, the percentages decrease to around 20% and 23.5%, respectively. Based on this, a percentage of approximately 20–25% can be used as the standard for the milling cutter to enter the stage of severe wear, providing a clear basis for determining the timing of milling cutter replacement. Alternatively, the threshold can be used as a warning value to reduce the frequency of workpiece quality inspection before approaching the threshold. It is advised to return to normal frequency when approaching the threshold to reduce the workload of quality inspection. In practical applications, the following expression can be used as a reference to determine the percentage threshold for replacement time:

(5) $$Percentage\;threshold=\left(0.25-0.05W_{p}\right)\times 100\%$$

where $W_{p}$ is a weight parameter with a value range of [0, 1].

The $W_{p}$ can be selected after considering the economy of the product and cutting tools, as well as the accuracy level of the product. When processing high-precision and high-value parts, more conservative parameters such as 0.1 can be selected to ensure machining accuracy and reduce scrap rates. When machining parts with low precision requirements or low cost, the parameters can be relaxed to 0.9 or 1 to improve the economy of the tool.

In addition, due to the different cutting parameters in the model’s testing and training datasets, the proposed method for determining the replacement time of worn milling cutters is suitable for different working conditions. Compared to the confusion matrix, which can only reflect the category of tool wear status, the wear transition percentage can better highlight the degradation process of tool wear throughout the entire life cycle, thus accurately selecting the timing of tool replacement.

### 4.3. Implementation Steps in Actual Production

When implementing the proposed method for determining tool replacement time in actual production, it is first necessary to collect full life spindle current data under several different cutting parameters. This step can be performed in parallel with normal production. There is no need to specifically arrange additional experiments to collect initial data for training neural networks. Then, the data from the first 20 surfaces and the last 20 surfaces must be used to create an SCCS image dataset. Afterwards, this dataset must be used to train a CNN model. Finally, the wear transition percentage can be calculated using the method discussed in Section 4.2 to determine the replacement time for worn milling cutters.

From the above-mentioned implementation steps, it can be seen that compared with other data-driven tool wear classification or regression methods, this method is easy to implement and does not require frequent stopping of machining during model training to measure tool wear. Only one Hall current sensor was used, and therefore, the cost of the device is relatively low. The mounting form of the sensor does not have an effect on the normal use of the machine. In addition, it has been tested that the calculation of the wear transition percentage on a regular industrial computer (Intel Celeron Processor N3060 (Intel, Santa Clara, CA, USA), without an independent GPU) takes less than 0.3 s, and therefore, this method has low computational requirements.

## 5. Discussion

The robustness of the method and the limitations of the current study are discussed in this section.

In order to evaluate the robustness of the method under different conditions, additional milling cutter wear life experiments (Experiment 2) were conducted. Similar to Experiment 1, this experiment also involved three cutting tools and three different machining parameters (C4, C5, and C6), as shown in Table 4. In addition, the workpiece material was TC4 titanium alloy, and the milling cutter material was carbide. The diameter of the milling cutters was 10 mm, and the number of teeth (edge) was four. The data preprocessing, dataset partitioning, and model training methods were the same as in Experiment 1.

**Figure 14:**
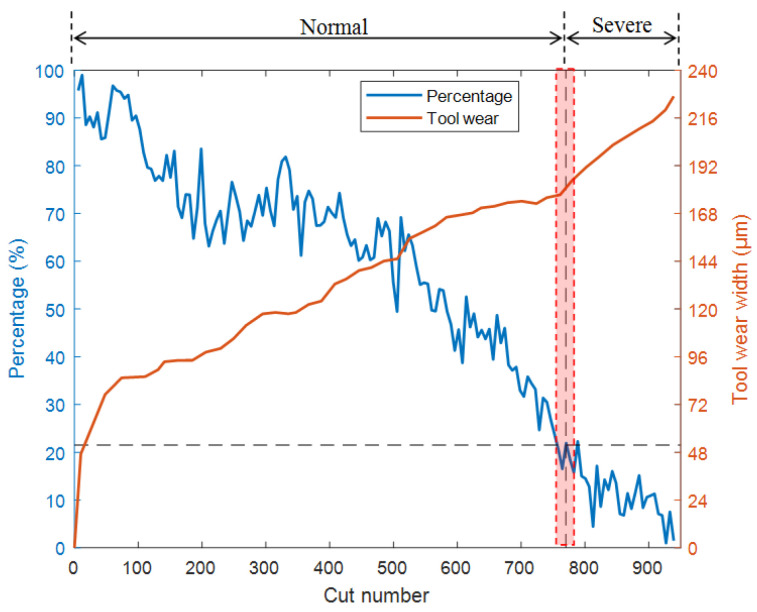
Test results of the T4 model.

The model T4 was obtained by training the model with the training datasets of C5 and C6. The T4 model was used to identify the full life data of C4 and calculate the wear transition percentage. The results are shown in Figure 14. As the tool wears, the wear transition percentage decreases continuously. When the percentage drops to around 21%, the tool state is in a transitional stage from normal wear to severe wear. The results are consistent with Experiment 1, demonstrating the robustness of the method under varying processing parameters.

In addition, the results of attempting to use the T4 model to identify the full life data of C1 were not satisfactory. This indicates that this method requires training a new model when the workpiece material or tool geometry is different. This is the main limitation of the current study.

The final indicator provided in this study for determining the timing of tool replacement is the wear transition percentage, which differs significantly from existing studies that only provide classification accuracy or regression error. At present, no similar research has been found, and therefore, direct comparison cannot be made. But it can be analyzed qualitatively. In terms of classification accuracy alone, using more complex models or models integrated with multiple methods will result in higher classification accuracy. However, the complexity and high computational cost of the model limit its practicality. The concept of wear transition percentage contains statistical ideas, and therefore, it does not require classifiers to have an absolute high accuracy. The work can be completed using a simple classification model, reducing computational requirements and having high practicality. The proposed method reduces costs on an acceptable basis, making it possible to deploy it on a large scale in factories.

## 6. Conclusions

Tool wear can reduce the accuracy and quality of products, so enterprises need to conduct tool health monitoring during production. However, the existing tool wear monitoring methods have high costs and complex operations, making them difficult to apply in practical production. To address this issue, a method for determining the timing of replacing worn milling cutters using SCCS and wear transition percentage is proposed. This method does not require stopping the machine to measure tool wear and is suitable for different machining parameters. Because the wear of cutting tools causes changes in the clutter components of the spindle current signal, this method uses a Vold–Kalman filter to remove the spindle rotation frequency and its harmonic components related to cutting parameters from the current. Subsequently, the SCCS with low correlation with cutting parameters is obtained. Then, a CNN is trained into a binary classification model using SCCS images from normal wear and severe wear stages. Finally, the trained CNN model is used to identify the full life SCCS data and calculate the wear transition percentage. By comparing with the measured wear width, the results show that when the wear transition percentage decreases to around 20–25%, the wear level is just in the transition stage from normal wear to severe wear. Therefore, the wear transition percentage can be used as an indicator for replacing milling cutters. The proposed tool wear monitoring method has a low cost and simple operation, making it suitable for large-scale deployment and application in factories. In addition, in future work, it is planned to extend this work to other machining processes, such as turning or drilling, and to explore solutions to the limitations of the current study.

## Figures and Tables

**Table 1:** Structural parameters of the CNN model.

No.	Layer Type	Kernel Size	Number of Kernels	Stride	Output
1	Input	-	-	-	160 × 160 × 1
2	Convolution	5 × 5	50	1 × 1	160 × 160 × 50
3	Pooling	4 × 4	50	4 × 4	40 × 40 × 50
4	Convolution	5 × 5	100	1 × 1	40 × 40 × 100
5	Pooling	8 × 8	100	8 × 8	5 × 5 × 100
6	Fully connected	-	-	-	60
7	Fully connected	-	-	-	10
8	Output	-	-	-	2

**Table 2:** Cutting parameters list.

Tool Symbol	Spindle Speed (r/min)	Feed Rate (mm/min)	*a_p_* (mm)	*a_e_* (mm)	Number of Completed Surfaces
C1	1200	540	5	2	116
C2	1200	828	4	2	156
C3	900	405	3	1	170

**Table 3:** Description of the datasets for training and testing models.

Model Symbol	Model Training		Determination of Replacement Timing
	Training Datasets	Testing Datasets	
T1	Training datasets of C2 and C3	Training datasets of C1	Full life datasets of C1
T2	Training datasets of C1 and C3	Training datasets of C2	Full life datasets of C2
T3	Training datasets of C1 and C2	Training datasets of C3	Full life datasets of C3

**Table 4:** List of cutting parameters for Experiment 2.

Tool Symbol	Spindle Speed (r/min)	Feed Rate (mm/min)	*a_p_* (mm)	*a_e_* (mm)	Number of Completed Surfaces
C4	1500	330	2	1	939
C5	1200	360	3	1	756
C6	1000	220	3	2	679

## Data Availability

The datasets generated in this study are available from the corresponding author upon reasonable request.
